# Choroidal Thickness in Eyes with Band Atrophy of the Optic Nerve from Chiasmal Compression

**DOI:** 10.1155/2022/5625803

**Published:** 2022-05-06

**Authors:** Luiz Guilherme Marchesi Mello, Ana Claudia F. Suzuki, Giuliana Rovito de Mello, Rony Carlos Preti, Leandro C. Zacharias, Mário L. R. Monteiro

**Affiliations:** ^1^Division of Ophthalmology and the Laboratory for Investigation in Ophthalmology (LIM-33), Faculdade de Medicina FMUSP, Universidade de São Paulo, São Paulo, Brazil; ^2^Department of Specialized Medicine, Centro de Ciências da Saúde (CCS), Universidade Federal do Espírito Santo, Vitória, Brazil

## Abstract

**Background:**

The choroid is a vascular tissue that helps maintain retinal and prelaminar optic nerve head function. Choroidal thickness has been previously studied in diseases accompanied by retinal neural loss, but the relationship between the two sets of measurements is not clear. In eyes with temporal hemianopia as a result of chiasmal compression lesions (CCL), retinal neural loss tends to be greater in the nasal than the temporal hemiretina, a fact that may be useful in evaluating the effect of inner retinal layer loss on choroidal thickness.

**Purpose:**

To evaluate macular and peripapillary choroidal thickness on swept-source optical coherence tomography (SS-OCT) in eyes with temporal hemianopia as a result of chiasmal compression and in healthy controls.

**Methods:**

33 eyes of 26 patients with band atrophy of the optic nerve and temporal visual field defects as a result of previously treated suprasellar tumors (CCL group) and 40 eyes of 21 healthy controls underwent SS-OCT scanning. The thickness of the peripapillary retinal nerve fiber layer (pRNFL), the peripapillary choroid (pChoroid), the macular RNFL (mRNFL), the macular ganglion cell layer (mGCL), and the macular choroid (mChoroid) was expressed globally and by sector (peripapillary quadrants and macular hemifield and quadrants). Ratios between macular nasal and temporal hemifield and quadrantic measurements were calculated using generalized estimated equation models, and the two groups were compared.

**Results:**

The pRNFL, mRNFL, and mGCC thicknesses were significantly smaller in the CCL group than in the control group (64.67 ± 10.53 *μ*m, 29.68 ± 5.80 *μ*m, and 80.60 ± 10.17 *μ*m *vs.* 103.78 ± 12.23 *μ*m, 39.89 ± 3.82 *μ*m, and 105.51 ± 7.76 *μ*m, respectively; *p* < 0.001). For the choroid, the only difference between the groups was increased macular nasal hemifield and superonasal quadrant thickness in CCL (222.47 ± 61.05 *μ*m and 230.45 ± 58.59 *μ*m in the CCL group, respectively *vs.* 190.68 ± 52.54 *μ*m and 197.65 ± 54.80 *μ*m in the control group, respectively; *p* < 0.05). The temporal/nasal ratios were significantly higher for the mRNFL and mGCC parameters and significantly lower for the mChoroid parameters in the CCL group, except for the superotemporal/superonasal quadrant ratio.

**Conclusions:**

The choroid does not thin after the inner retinal layer becomes damaged due to CCL and may even be thicker in some areas with corresponding severe retinal neural loss. While further studies are needed to interpret these findings, choroidal thinning is most likely not secondary to optic nerve disease-related inner retinal neural loss.

## 1. Introduction

The choroid plays an important role in the maintenance of ocular function. Several retinal diseases are known to be related to choroidal thickness abnormalities and vice versa [1]. The peripapillary choroid (pChoroid) is believed to supply the prelaminar region of the optic nerve head and is possibly associated with peripapillary retinal nerve fiber layer (pRNFL) nutrition and optic nerve head damage [2]. New noninvasive imaging techniques, such as enhanced depth imaging and swept-source optical coherence tomography (SS-OCT), have done much to improve the assessment of morphological features of the choroid [1].

Several ocular and systemic diseases (e.g., mitochondrial disease, migraine, multiple sclerosis, and autoimmune disease) are associated with choroidal and retinal thinning [3–6]. Choroid thinning in glaucomatous eyes was found to be associated with retinal nerve fiber layer (RNFL) atrophy and visual field (VF) defects in some studies [7–10] but not in others [11–14]. Studies in patients with Leber's hereditary optic neuropathy and dominant optic atrophy suggest that thinning of the macular ganglion cell layer (mGCL) and the RNFL can lead to choroidal thinning [6, 15], but it remains unclear whether choroidal thinning is secondary to retinal ganglion cell/RNFL loss or due to disease-related vascular abnormalities.

Chiasmal compressive lesions (CCL) constitute a potentially important model for the evaluation of the effect of inner retinal layer loss on functional parameters and other eye structures [16–18]. This type of anterior visual pathway damage usually causes temporal hemianopia as a consequence of axonal damage from the crossed RNFL in the chiasm (originating from the nasal hemiretina), leading to band atrophy (BA) of the optic nerve head (a peripapillary RNFL thinning and pallor of the optic disc predominantly in temporal and nasal sectors), and loss of retinal ganglion cells and RNFL in the nasal hemiretina [19]. The fact that lesions occur at the chiasm, with no indication of primary choroidal abnormality, make it an appropriate model for evaluating the effect of inner retinal neural loss on the choroid.

To explore these possibilities, we measured peripapillary and macular choroidal thickness on SS-OCT in patients with CCL and temporal visual field (VF) defects and in healthy controls. The macular RNFL (mRNFL), macular GCL (mGCL), and macular choroid (mChoroid) parameters included global average, quadrant, and hemisector thickness. The corresponding pRNFL and pChoroid parameters were global average and quadrant thickness.

## 2. Materials and Methods

33 eyes of 26 patients with CCL as a result of previously treated suprasellar tumors and 40 eyes of 21 healthy controls were included in this prospective, cross-sectional study. Chiasmal compression was confirmed on MRI at the time of diagnosis, followed by effective decompression and stable VF defects for at least 6 months before study entry. The inclusion criteria were best-corrected visual acuity (VA) ≥20/30, refractive error within ±5 diopters, and intraocular pressure <21 mmHg. Exclusion criteria were media opacities, other optic neuropathies, retinal diseases, optic disc abnormalities, alcohol/tobacco abuse, diabetes mellitus, arterial hypertension treated with more than three antihypertensive drugs, and other conditions that could affect the choroid. The study protocol complied with the Declaration of Helsinki and was approved by the Institutional Review Board Ethics Committee (CAAE 69238217.0.0000.0068). All participants gave their informed written consent.

### 2.1. Ophthalmological Examination

All patients underwent ophthalmological examination, including VA assessment (measured with a standard Snellen chart at 6 meters), slit-lamp biomicroscopy, Goldmann applanation tonometry, fundoscopy, VF, and SS-OCT. VF was assessed with the 24-2 SITA-Standard strategy on standard automated perimetry (Humphrey Field Analyzer; Carl Zeiss Meditec, Dublin, CA) using a Goldmann size III stimulus. The reliability criteria included fixation loss ≤20%, false-positive rate ≤15%, and false-negative rate ≤30%. CCL eyes were required to have partial or complete temporal hemianopia and a nasal hemifield within normal limits (absence of clusters of ≥3 points with *p* < 5% on the pattern deviation plot).

**Figure 1:**
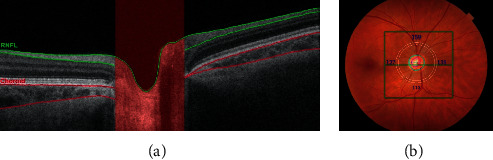
Choroidal and retinal nerve fiber layer (RNFL) evaluation of a right optic disc. (a) Optical coherence tomography (OCT) showing a B-scan in the peripapillary area with the RNFL and choroidal layer delimited in green and red, respectively. (b) Fundus photography of the right eye and the peripapillary area of OCT acquisition delimited by a white circle centered on the optic nerve head and divided into four sectors (temporal, superior, nasal, and inferior).

**Figure 2:**
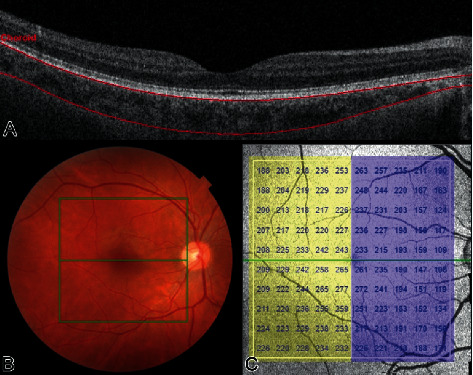
Choroidal evaluation of a right macula. (a) Optical coherence tomography (OCT) of showing a B-scan at the fovea with the choroidal layer outlined in red. (b) Fundus photography of the right eye and the macular area of OCT acquisition delimited in green. (c) Posterior pole map of the right eye divided into nasal (blue) and temporal (yellow) hemiretina and columns from 1 to 10. In the left eye, the columns maintain the sequence from temporal to nasal, inverting the order of the numbers (10 to 1).

After the VA and VF assessment, the pupils were dilated with 1% tropicamide eye drops for fundus examination, and an SS-OCT scan (DRI OCT Triton Plus®V.10.11., Topcon, Japan) was performed in the morning to avoid diurnal variation in choroidal thickness. A 6 × 6 mm area centered on the optic disc and a 7 × 7 mm area centered on the fovea were scanned. Only images with signal intensity >40 and without artifacts were analyzed. The SS-OCT software (IMAGEnet^®^ 6 V.1.21.11783) automatically segmented the pRNFL, the pChoroid, and the macular retinal layers and choroid. The following macular thickness parameters were recorded: mRNFL, ganglion cell layer + inner plexiform layer (mGCL+), the sum of mRNFL and mGCL+ (referred to as the macular ganglion complex or mGCC), and the mChoroid. The peripapillary area was considered to be a circular region (*Ø* = 3.4 mm) around the optic nerve head. pRNFL and pChoroid thicknesses were expressed as the global average (360°) and by sector (temporal 310-41°, superior 41–120°, nasal 121–230°, and inferior 231–310°) (Figure 1). The OCT software subdivided the macula into 100 squares to calculate the global average, nasal and temporal hemiretinal thickness (50 squares each), and quadrant thickness: superotemporal (ST), inferotemporal (IT), superonasal (SN), and inferonasal (IN). We also calculated the temporal/nasal hemifield ratio (T/N) and mirrored quadrant ratios (ST/SN and IT/IN) (Figure 2).

### 2.2. Data Analysis and Statistics

The descriptive statistics consisted of mean values ± SD. The Kolmogorov–Smirnov test was used to evaluate the normality assumption of the data. Groups were compared using generalized estimating equation (GEE) models to compensate for the use of both eyes from the same subject. A *p* value of <0.05 was considered statistically significant. All statistical analyses were performed using the software IBM SPSS Statistics v. 25.

## 3. Results

33 eyes of 26 patients with CCL (24 from pituitary adenoma and 2 from craniopharyngiomas) and 40 control eyes (CT) were studied (Table 1). No statistically significant difference was observed between the groups with regard to age and spherical equivalent. VA and VF mean deviation (MD) were greater in the CCL group than in the CT group. In the CCL group, 16 eyes presented complete temporal hemianopia, 6 had VF defects in approximately one quadrant, and 11 had defects in less than one quadrant.

All pRNFL thickness parameters were significantly smaller in the CCL group than in the CT group, but the groups did not differ with regard to pChoroid parameters (Table 2). In the macular evaluation (Table 3), CCL eyes had significantly thinner mRNFL and mGCC, both globally and the sectors. Furthermore, all mRNFL and mGCC ratios (temporal vs. nasal) were higher in the CCL group. Most of the choroidal parameters in the macular area (global average, temporal hemifield, ST, IT, and IN) were statistically similar, but the nasal hemiretina and SN were significantly thicker in CCL than in CT. All mChoroid ratios (temporal vs. nasal) were smaller in the CCL group, although one (ST/SN) did not reach statistical significance.

## 4. Discussion

Optic pathway lesions as a result of chiasmal compression may be used as a model to investigate the effect of isolated retinal neural loss on other ocular structures [18, 19]. In eyes with resolved CCL and remaining temporal VF defects, a marked difference was observed between the severely affected nasal hemiretina and the relatively preserved temporal hemiretina. In this model, OCT is a useful noninvasive tool for demonstrating neuronal damage, as shown in the present study (pRNFL, mRNFL, and mGCC sectors were thinner in CCL than in CT). Furthermore, it can also reveal deeper retinal abnormalities secondary to CCL, such as thickening of the inner nuclear layer, outer plexiform layer, and photoreceptor layer [18]. New technologies such as OCT angiography and SS-OCT have greatly facilitated the assessment of retinal vessel morphology [16] and deeper ocular tissues such as the choroid. Thus, a recent study found that reduced peripapillary and macular vessel densities in eyes with CCL were correlated with retinal neural loss and visual field damage [16]. Therefore, the model may be useful in investigating the effect of retinal neural loss in the choroid.

The choroid is essentially a vascular tissue directly related to the retina and the anterior portion of the optic nerve. Researchers evaluating other ocular diseases that typically progress with axonal damage of the retinal ganglion cells have reported choroidal thinning in eyes with RNFL loss [6–10, 15]. Choroidal thinning might be the result of decreased metabolic activity that is associated with the atrophy of retinal ganglion cells [20]. One study retrospectively evaluated the choroid after traumatic optic neuropathy by comparing the traumatized eye to the unaffected contralateral eye [21] and observing increased choroidal thickness in the former. However, the nature of the lesion (blunt or high-energy trauma) and the timing of the OCT scan (two weeks) should be considered when interpreting the data. Since previous studies showed that at least 5 weeks were required to reduce pRNFL and macular thickness after a traumatic indirect optic nerve injury [22], it is unlikely that the study was able to fully assess the effect of inner retina atrophy on the choroid thickness.

To our knowledge, this is the first study to evaluate choroidal thickness in patients with CCL. Nevertheless, no direct association was found between choroidal thinning and CCL in a setting of exclusive RNFL loss. Interestingly, in eyes with CCL, the nasal macular choroidal sector displayed increased thickness, while the other parameters (the temporal macular sector, the global average, and the peripapillary area) were statistically similar in the two groups. Although not all sectors in CCL eyes were significant, the choroid was generally thicker in the macular and peripapillary areas. The posterior ciliary arteries are the main blood suppliers to the optic nerve head and choroid [23]. It is reasonable to assume that RNFL and GCL loss reduces the metabolic demand of the retina and optic nerve head, changes the blood flow, decreases vessel density, and affects choroidal circulation. Furthermore, some studies found a direct correlation between macular choroidal thickness and the thickness of the outer retinal layers [24, 25]. Therefore, increased thickness of the outer retinal layers in CCL eyes, as previously described by de Araujo et al. [18], might also have influenced choroidal measurements in our study. However, more research on the structural and functional characteristics of the choroid and larger sample studies are needed to clarify the choroidal changes observed in CCL. Despite some limitations, such as a relatively small sample size and possible subclinical damage in the temporal retina despite a normal nasal VF in the CCL group [18], our study found no association between exclusive RNFL damage as a result of CCL and choroidal thinning. This finding may be useful in the evaluation of patients with compressive lesions of the anterior visual pathway and may, to some degree, be generalizable to choroidal abnormalities in other diseases.

## 5. Conclusions

The choroid does not thin after the inner retinal layer becomes damaged due to CCL and may even be thicker in some areas with corresponding severe retinal neural loss. In diseases with features related to RNFL and choroidal thinning, it is important to investigate other direct mechanisms of choroidal damage. While further studies are needed to interpret our findings, choroidal thinning is most likely not secondary to optic nerve disease-related inner retinal neural loss.

## Figures and Tables

**Table 1:** Demographic data of study participants.

	CCL	CT	*p* value
Subjects, *n*	26	21	
Eyes studied	33	40	
Sex (F : M)	11 : 15	13 : 8	
Age, y, mean (SD)	47.84 (2.53)	48.75 (2.87)	0.812^*∗*^
VA, mean (SD)	0.96 (0.01)	1.0 (0.01)	**0.003** ^*∗*^
SEQ, mean (SD)	−0.24 (0.25)	0.19 (0.28)	0.253^*∗∗*^
MD, mean (SD)	−9.35 (0.64)	−0.76 (0.62)	**<0.001** ^*∗∗*^

CCL = chiasm compressive lesions group; CT = control group; F = female; M = male; MD = mean deviation of the 24-2 visual field; *n* = number of subjects; SD = standard deviation; SEQ=spherical equivalent; VA = best-corrected visual acuity; y = years. ^*∗*^Student's *t*-test. ^*∗∗*^Generalized estimated equations. Significant values are in bold.

**Table 2:** Mean values (±standard deviation) of peripapillary retinal nerve fiber layer and choroidal thickness measurements (*μ*m) in eyes with temporal hemianopia from chiasmal compressive lesions (CCL) and healthy controls (CT).

Parameter		CCL	CT	*p* value^*∗*^
pRNFL	Global average	64.67 (10.53)	103.78 (12.23)	**<0.001**
	Temporal quadrant	39.36 (10.87)	76.83 (11.38)	**<0.001**
	Nasal quadrant	42.52 (8.57)	82 (14.79)	**<0.001**
	Superior quadrant	90.45 (18.38)	126.11 (15.20)	**<0.001**
	Inferior quadrant	97.83 (16.73)	141.72 (22.76)	**<0.001**

pChoroid	Global average	156.42 (52.30)	137.83 (43.94)	0.188
	Temporal quadrant	159.00 (54.16)	135.55 (46.62)	0.740
	Superior quadrant	173.67 (60.50)	156.65 (51.01)	0.275
	Nasal quadrant	156.61 (51.10)	140.20 (44.83)	0.288
	Inferior quadrant	136.00 (57.81)	120.55 (42.18)	0.283

pRNFL = peripapillary retinal nerve fiber layer; pChoroid = peripapillary choroid. ^*∗*^Generalized estimated equations. Significant values are in bold.

**Table 3:** Mean values (±standard deviation) of inner macular and choroid thickness parameters in eyes with temporal hemianopia from chiasmal compressive lesions (CCL) and healthy controls (CT).

Parameter^*∗*^		CCL	CT	*p* value^∗∗^
mRNFL thickness (*μ*m)	Global average	29.68 (5.80)	39.89 (3.82)	**<0.001**
	Temporal hemiretina	24.39 (3.40)	26.83 (2.03)	**<0.001**
	Nasal hemiretina	34.98 (8.57)	52.95 (6.06)	**<0.001**
	STQ	23.34 (3.46)	25.93 (2.53)	**<0.001**
	ITQ	25.44 (3.70)	27.73 (2.58)	**0.003**
	SNQ	30.85 (8.14)	50.73 (5.48)	**<0.001**
	INQ	39.10 (9.74)	55.17 (7.44)	**<0.001**

mRNFL ratios	T/N	0.72 (0.11)	0.51 (0.04)	**<0.001**
	STQ/SNQ	0.78 (0.14)	0.51 (0.05)	**<0.001**
	ITQ/INQ	0.67 (0.12)	0.51 (0.05)	**<0.001**

mGCC thickness (*μ*m)	Global average	80.60 (10.17)	105.51 (7.76)	**<0.001**
	Temporal hemiretina	81.61 (9.68)	92.43 (6.90)	**<0.001**
	Nasal hemiretina	79.59 (11.51)	118.59 (9.28)	**<0.001**
	STQ	79.49 (8.82)	91.75 (7.23)	**<0.001**
	ITQ	83.74 (9.82)	93.11 (6.95)	**<0.001**
	SNQ	76.64 (11.80)	117.59 (9.42)	**<0.001**
	INQ	82.54 (11.94)	119.59 (9.77)	**<0.001**

mGCC ratios	T/N	1.03 (0.08)	0.78 (0.03)	**<0.001**
	STQ/SNQ	1.04 (0.09)	0.78 (0.03)	**<0.001**
	ITQ/INQ	1.02 (0.08)	0.78 (0.04)	**<0.001**

mChoroid thickness (*μ*m)	Global average	233.63 (53.58)	212.68 (48.26)	0.102
	Temporal hemisector	244.79 (50.21)	234.68 (49.34)	0.355
	Nasal hemisector	222.47 (61.05)	190.68 (52.54)	**0.036**
	STQ	253.86 (54.88)	236.81 (55.12)	0.147
	ITQ	235.71 (57.20)	232.56 (50.75)	0.829
	SNQ	230.45 (58.59)	197.65 (54.80)	**0.023**
	INQ	214.50 (66.75)	183.71 (53.91)	0.070

mChoroid ratios	T/N	1.13 (0.17)	1.27 (0.21)	**0.020**
	STQ/SNQ	1.13 (0.20)	1.23 (0.21)	0.121
	ITQ/INQ	1.13 (0.18)	1.31 (0.25)	**0.005**
	Columns 5/6	1.02 (0.04)	1.05 (0.04)	**0.002**
	Columns 4/7	1.06 (0.12)	1.15 (0.14)	**0.003**
	Columns 3/8	1.13 (0.18)	1.29 (0.26)	**0.003**
	Columns 2/9	1.22 (0.28)	1.45 (0.41)	**0.005**
	Columns 1/10	1.32 (0.39)	1.63 (0.60)	**0.008**

INQ = inferonasal quadrant; ITQ = inferotemporal quadrant; mChoroid = macular choroid; mGCC = macular ganglion cell complex; mRNFL = macular retinal nerve fiber layer; SNQ=superonasal quadrant; STQ = superotemporal quadrant; T/N = temporal average/nasal average ratio. ^*∗*^For analysis purposes, the square macular area was divided into temporal and nasal hemisectors, which were further divided into superior and inferior quadrants. ^*∗∗*^Generalized estimated equations. Significant values are in bold.

## Data Availability

The data are available from the corresponding author upon request.
